# Molecular characteristics of *Neisseria meningitidis* carriage strains in university students in Lithuania

**DOI:** 10.1186/s12866-023-03111-5

**Published:** 2023-11-17

**Authors:** Inga Ivaškevičienė, Justina Silickaitė, Asta Mačionienė, Rimvydas Ivaškevičius, Aistė Bulavaitė, Vilmantas Gėgžna, Silvija Kiverytė, Božena Paškevič, Aurelija Žvirblienė, Milda Plečkaitytė

**Affiliations:** 1 Clinic of Children’s Diseases, Institute of Clinical Medicine, Faculty of Medicine, Vilnius University, Vilnius, Lithuania; 2 Pediatric Center, Vilnius University Hospital Santaros Klinikos, Vilnius, Lithuania; 3 Institute of Biotechnology, Life Sciences Center, Vilnius University, Vilnius, Lithuania; 4 Center of Laboratory Medicine, Vilnius University Hospital Santaros Klinikos, Vilnius, Lithuania; 5 Institute of Biosciences, Life Sciences Center, Vilnius University, Vilnius, Lithuania; 6 Department of Physiology, Biochemistry, Microbiology and Laboratory Medicine Institute of Biomedical Sciences, Faculty of Medicine, Vilnius University, Vilnius, Lithuania

**Keywords:** *Neisseria meningitidis*, Oropharyngeal carriage, Invasive meningococcal disease, Serogroup B, Vaccine antigens, Vaccines, Risk factors

## Abstract

**Background:**

*Neisseria meningitidis* can be carried asymptomatically in the human oropharynx without causing symptoms. Meningococcal carriage is relevant to the epidemiology of invasive meningococcal disease (IMD). No carriage studies have been performed among the general population in Lithuania, whereas the incidence of IMD in Lithuania was among the highest in European countries from 2009 to 2019.

**Results:**

We analyzed a total of 401 oropharyngeal samples collected from university students from December 2021 to February 2023 for *N. meningitidis* carriage using direct swab PCR assays and culture. The overall carriage prevalence based on both or either swab PCR or culture was 4.99%. PCR-based assays were used to characterize 15 carriage isolates, including detection of genogroup, multilocus sequence typing profile, and typing of antigens PorA and FetA. The most common carriage isolates were capsule null locus (*cnl*), accounting for 46.7%, followed by genogroups B (26.7%) and Y (13.3%). We also performed a molecular characterization of invasive *N. meningitidis* isolates collected during the COVID-19 pandemic and post-pandemic period to understand better the meningococcal carriage in the context of prevailing invasive strains. Despite the substantial decrease in the incidence of IMD during the 2020–2022 period, clonal complex 32 (CC32) of serogroup B continued to be the most prevalent IMD-causing CC in Lithuania. However, CC32 was not detected among carriage isolates. The most common CCs were CC269, CC198, and CC1136. The antigen peptide variants found in most carried isolates were classified as ‘insufficient data’ according to the MenDeVAR Index to evaluate the potential coverage by the 4CMenB vaccine. Nearly half of the isolates were potentially covered by the Men-Fhbp vaccine. Resistance to ciprofloxacin was detected only for one *cnl* isolate. All isolates were susceptible to penicillin and ceftriaxone. Our analysis identified frequent partying (≥ 4 times/month) as a risk factor for meningococcal carriage, whereas smoking, living in a dormitory, and previous COVID-19 illness were not associated with the carriage.

**Conclusions:**

Our study revealed a low prevalence of meningococcal carriage among university students in Lithuania. The carriage isolates showed genetic diversity, although almost half of them were identified as having a null capsule locus.

**Supplementary Information:**

The online version contains supplementary material available at 10.1186/s12866-023-03111-5.

## Introduction

*Neisseria meningitidis* is a human-specific bacterium that is often asymptomatically carried in the nasopharynx in up to 35% of individuals at any given time [1, 2]. The prevalence of meningococcal carriage varies by age with the highest carriage rates found among 16-24-year-olds in industrialized countries [3–5]. Risk factors for meningococcal carriage include living in overcrowded places, smoking, attending bars/pubs, and other types of social mixing [6, 7]. The age group with a high carriage prevalence is considered an important transmitter of *N. meningitidis*. Transmission occurs through respiratory secretions resulting from close contact among individuals. It is not fully understood why meningococci in some colonized individuals may pass the mucosal barrier and enter the bloodstream causing sepsis and meningitis [1, 8]. Invasive meningococcal disease (IMD) is a rapidly evolving condition with significant complications and poor outcomes, especially in children and young adults. Most cases of IMD worldwide are caused by six serogroups (A, B, C, W, Y, and Z) [9], but the distribution of the serogroup and the incidence of IMD vary by geographic location and economic development of the region/country [2, 10].

The application of molecular techniques to characterize *N. meningitidis* isolates revealed that clonal complexes (CCs) associated with IMD comprise only a fraction of carriage isolates [5, 11, 12]. CCs of carriage isolates are more heterogeneous, whereas IMD is caused by a limited number of CCs. Many carried isolates lack the capsule, which may facilitate their colonization of the human nasopharynx and evasion of the host immune system [13]. The character of IMD occurrence suggests that meningococcal carriage is relevant to the epidemiology of the disease, but the relationship between carriage and disease is not yet fully understood [1, 2, 8]. It was demonstrated that co-colonization with pathogenic meningococcal strains can occur during the carrier state, thereby raising the potential for recombination and subsequent capsule switching [14]. Vaccination is an effective factor of preventing IMD and may also help to reduce *N. meningitidis* oropharyngeal carriage and prevent its transmission. A previous study in the UK demonstrated a significant reduction in oropharyngeal carriage of genogroups W and Y meningococci following the introduction of the conjugate MenACWY vaccine. Carriage of genogroup B meningococci remained unchanged, while a consistently low carriage of genogroup C was observed [15]. Studies in several countries did not find evidence indicating an impact of the 4CMenB vaccine on the prevalence of genogroup B carriage and other disease-causing genogroups [15, 16].

The incidence of IMD in Lithuania was one of the highest in European countries during the past two decades with an average of 2.24 cases/100,000 population [17, 18]. Serogroup B (MenB) was the leading cause of IMD. Recent molecular analysis of invasive isolates collected from 2009 to 2019 in Lithuania revealed that CC32, CC41/44, and CC18 were the most common CCs, with MenB strain P1.19,15: F4-28: ST-34 (CC32) accounting for 64.1% of recovered IMD isolates [18]. Due to COVID-19 restrictive measures, the incidence of IMD decreased from 1.3 cases per 100,000 population in 2019 to 0.43 cases and 0.39 cases per 100,000 in 2020 and 2021, respectively. The incidence remained low in 2022 at 0.46 cases per 100,000 individuals [17].

In 2018, the four-component meningococcal B vaccine (4CMenB) was introduced into the nationally funded vaccination program with a recommendation for use in preventing IMD for individuals from 3 months of age. The two-component meningococcal B vaccine (MenB-Fhbp) is authorized for the vaccination of individuals 10 years and older, but the cost of this vaccine is not covered by the national reimbursement system.

No studies have been conducted on the carriage of *N. meningitidis* in Lithuania among the general population.

We carried out a meningococcal carriage study among university students (aged 18–25 years) in Vilnius, the largest city in Lithuania, located in the region most affected by IMD. The goal of the study was to determine the prevalence of *N. meningitidis* throat carriage and to characterize carried meningococcal isolates. Additionally, our objective was to identify risk factors for carriage in this age group. We also performed a molecular characterization of invasive meningococcal isolates collected throughout the COVID-19 pandemic and the post-pandemic period (2020–2022) to improve our understanding of *N. meningitidis* carriage in relation to the predominant invasive meningococcal strains.

## Methods

### Study design and study subjects

The study was carried out in Lithuania from December 2021 to February 2023. Healthy participants aged 18–25 years from a single university situated in the Vilnius region were invited to take part in this study on a voluntary basis. All participants were Caucasian Lithuanians. The inclusion criteria were living and studying in the Vilnius region for at least four weeks before enrollment. Exclusion criteria included prior vaccination against meningococci, antibiotic treatment four weeks before sampling, and acute upper respiratory tract infection during enrolment. The participants completed a questionnaire that included questions about their age, gender, residency in either a dormitory or non-dormitory accommodation, smoking habits, social life, and any previous history of COVID-19 disease.

### Sample collection

An oropharyngeal swab was collected from the posterior pharynx by trained medical personnel. The swabs were then placed into sterile vials containing 1 mL of Amies medium (Eswab, Copan, Brescia, Italy). The samples were stored at room temperature and transported to the Laboratory of Microbiology at Vilnius University Hospital Santaros Klinikos within two hours for immediate microbiological processing.

### Isolation of *N.meningitidis*

Fifty-µL of the specimen was cultured on Thayer-Martin agar medium (Oxoid, UK) for 48–72 h at 35°C and 5% CO_2_. Candidate *N. meningitidis* was subcultured on Chocolate agar (Oxoid, UK) and identified using matrix-assisted laser desorption ionization time-of-flight mass spectrometry (MALDI-TOF MS, Bruker Daltonics, Bremen, Germany). At least three colonies of each meningococcal isolate were selected for further analysis. Heat-inactivated extracts of pure isolates were prepared for analysis by PCR.

### *N. meningitis* identification in oropharyngeal swabs

Genomic DNA was extracted from 500 µL of the specimen using a GeneJET genomic DNA purification kit (Thermo Fisher Scientific, Vilnius, Lithuania) according to the manufacturer’s instructions. Real-time PCR (rt-PCR) assays were performed using TaqMan primers targeting *ctrA* [19] and *porA* [20]. The RNAse P target was amplified in rt-PCR reaction (Applied Biosystems, Thermo Fisher Scientific) as a positive control for the DNA extraction method. Samples with Ct values < 35 were considered positive and Ct values > 40 were considered negative [19, 20]. Samples with Ct values 35–40 were repeatedly assayed using 4- and 10-fold diluted DNA to reveal the suspected inhibition of PCR.

### Molecular characterization of *N. meningitidis* carriage isolates

Genogrouping was performed using a singleplex polymerase chain reaction (PCR)-based assay, as described recently [18]. Genogroup E was detected as described in [21]. For isolates that tested negative for genogroup, the intergenic region between the regions D and E of the *cps* cluster was amplified using previously described primers GH26R (5’-GGTCGTCTGAAAGCTTGCCTTGCTC-3’) and HC287 (3’-CGCGCCATTTCTTCCGCC-3’) or HC344 (5’-GGATTGGACGAGCGAGAC-3’) [22] to detect the capsule null locus (*cnl*). In cases where *cnl* amplification did not yield a PCR product, the presence of *ctrA* was tested using primers described in [23].

MLST, PorA, and FetA typing were performed following the protocol described on the PubMLST Neisseria website [24] and detailed in [18]. Allele assignment and multilocus sequence typing (MLST) sequence type (ST) and CC were obtained from the PubMLST Neisseria database [24].

The genes coding for vaccine antigens fHbp, NHBA, and NadA were amplified using previously described primers [25] and PCR conditions [18]. For *fHbp*-negative isolates, the absence of the *fHbp* gene was verified using multiple PCR assays, including primers described in [26, 27], primers targeting sequences within flanking genes (1869-2F and 1871-Ralt) [28], and an alternative reverse primer (gna1870R2) due to a deletion downstream of the *fHbp* gene [29] (Additional file 1).

Sequence data from the antigen gene sequencing was analyzed to determine the alleles and peptide variants using the PubMLST Neisseria database [24]. The MenDeVAR Index method was used to estimate the predicted coverage of the 4CMenB and MenB-Fhbp vaccines [30]. The Index provides information on the reactivity of *N. meningitidis* antigens to MenB vaccines, such as factor H binding antigen (fHbp), Neisserial heparin binding antigen (NHBA), and Neisseria adhesin A (NadA). The degree of antigen reactivity was expressed as an ‘exact match’ to meningococcal variants with one or more exact sequence matches to vaccine variants (‘green’ Index); ‘cross-reactive’ to meningococci with one or more cross-reactive antigenic variant (‘amber’ Index); ‘not-reactive’ to meningococci where all antigenic variants were neither exact matches nor demonstrated cross-reactivity to vaccine variants (‘red’ Index). Antigenic variants that were not tested in experimental assays were labeled ‘insufficient data’ (‘grey’ Index).

### Antibiotic susceptibility testing of *N. meningitidis* carriage isolates

Susceptibility to penicillin, ceftriaxone, and ciprofloxacin was determined by MIC test (Liofilchem, Italy) on Mueller-Hinton agar with 5% sheep blood (Graso Biotech, Poland). Minimal inhibitory concentration (MIC) breakpoints were interpreted according to EUCAST [31].

### Molecular characterization of invasive *N. meningitidis* isolates collected from 2020 to 2022

In Lithuania, all cases of IMD are required to be reported according to the national guidelines [32]. Since 2009, invasive *N. meningitidis* isolates have been requested to be sent to the National Public Health Surveillance Laboratory (NPHSL). The NPHSL received a total of 20 invasive isolates between 1 January 2020 and 31 December 2022. Two isolates collected in 2021 could not be cultured, so they were excluded from the study. Two isolates obtained in 2022 were from the same individual, therefore only one isolate was included. Information on the gender, age, and region of isolation was obtained from the NPHSL. The study analyzed a total of 17 invasive isolates (n = 6, 2020; n = 6, 2021; n = 5, 2022) that were recovered from the blood or cerebrospinal fluid of individuals with IMD. Meningococcal isolates were serogrouped by slide agglutination at the NPHSL. In this study, genogrouping was performed using a singleplex polymerase chain reaction (PCR)-based assay [18].

Whole genome sequencing (WGS) and genome assembling of the isolates collected in 2020 (n = 6) were performed using funding from the European Centre for Disease Prevention and Control (Solna, Sweden). The WGS was conducted by Eurofins Genomics Europe Sequencing Gmbh (Konstanz, Germany) using the Illumina NovaSeq platform, and assembly was performed using SPAdes 3.11 [33] with careful mode enabled. Genome contigs were submitted to the PubMLST Neisseria database [24]. The allelic profile of seven MLST genes and variable regions of PorA and FetA were determined by automatic scanning of genome contigs [24]. For isolates that were not subjected to WGS (2021–2022), capsular genogrouping, MLST, PorA, and FetA typing was performed as described for *N. meningitidis* carriage isolates.

### Statistical analysis

Data were analyzed using the R statistical software, version 4.3.0 [34], with additional packages DescTools 0.99.48 [35], gtsummary 1.7.0 [36], broom 1.0.4 [37], and Microsoft Excel. 95% confidence intervals (CIs) of proportions were calculated using Wilson’s method [38] implemented in the R package DescTools and then converted to percentages. Logistic regression was used to examine the association between selected explanatory factors (candidate risk factors) and the status of *N. meningitidis* carriage. Univariate (unadjusted) and multivariate (adjusted for possible confounders) analyses were performed as described in [7]. The results were presented as odds ratios (OR) of the reference category versus each remaining category, along with their corresponding 95% confidence intervals and two-sided *p*-values. Statistically significant results were defined as *p*-value < 0.05. All potential risk factors considered for meningococcal carriage were analyzed as categorical variables.

## Results

### Molecular characterization of invasive meningococcal isolates recovered between 2020 and 2022

Between 2020 and 2022, a total of 36 cases of IMD were reported in Lithuania, resulting in an overall incidence rate of 0.43 cases per 100,000 individuals [17]. Seventeen invasive isolates analyzed in this study accounted for 47.2% of all reported IMD cases during the same period. An isolate was identified as belonging to serogroup X (collected in 2022), while the remaining 16 isolates were of MenB. The isolates were grouped into 10 STs, 8 of which were only detected once (Table 1). Three of the six new STs were grouped into three CCs, while the others were not linked to any CC (UA). The isolates were assigned to three CCs, with CC32 being the most common, accounting for 64.7% (n = 11) of all isolates (50% (n = 3) in 2020; 83% (n = 5) in 2021; 60% (n = 3) in 2022). Antigen finetyping revealed that the MenB strain P1.19,15: F4-28: ST-34 (CC32) represented 41.2% (n = 7) of all recovered meningococcal isolates.

**Table 1:** Molecular characteristics of invasive meningococcal isolates recovered in Lithuania, 2020–2022

Genogroup	Sequence type	Clonal complex	No. of isolates
B	34	32	7
B	9775	32	1
B	9779	32	1
B	16791	32	1
B	16805	32	1
B	213	213	1
B	17490	461	1
B	15967	UA	2
B	16806	UA	1
X	17491	UA	1

### Characteristics of study participants

Remote teaching and learning were introduced into the Lithuanian educational system due to COVID-19. However, as of 1 September 2021, most Lithuanian higher education institutions and universities had authorized contact lectures and other teaching activities. The study enrolled a total of 405 university students. Four participants were excluded because their samples tested negative for RNAase P in rt-PCR. Among the 401 included participants, 50 (12.5%) were recruited in December 2021, 290 (72.3%) in 2022, and 61 (15.2%) between January and February 2023. Out of 401 participants, 91 (22.7%) were male and 310 (77.3%) were female. The median age was 22.4 (range 18–25 years).

### *N. meningitidis* carriage prevalence

If found to be positive for both or either *ctrA* or *porA* by direct swab PCR assays, samples were considered *N. meningitidis* positive. There was no 1:1 match between swab PCR and culture-detected meningococci (Table 2). Five samples detected as negative for *N. meningitidis* by direct swab PCR assays were culture positive. Five samples were culture negative but positive by PCR assays. Ten samples were tested positive by both culture and PCR (Table 2).

**Table 2:** * N. meningitidis* PCR and culture results for oropharyngeal swabs and genogrouping of carried isolates recovered in Lithuania, December 2021–February 2023

Swab no.	Direct oropharyngeal swab PCR		Culture	Isolate PCR		Genogroup designation
	*ctrA*	*porA*		*ctrA*	Genogroup specific gene	
NM-028	−	−	+	−	−	cnl
NM-036	+	+	+	+	+	B
NM-038	−	+	−	−	−	−
NM-059	−	+	+	−	−	cnl
NM-063	−	+	+	−	−	cnl
NM-089	+	+	+	+	+	Y
NM-110	−	+	+	−	−	cnl
NM-115	−	−	+	+	−	NG
NM-120	−	+	−	−	−	−
NM-138	−	+	−	−	−	−
NM-164	−	+	−	−	−	−
NM-168	+	+	+	+	−	NG
NM-176	+	+	+	+	+	B
NM-201	+	+	+	+	+	B
NM-212	−	+	−	−	−	−
NM-253	−	+	+	−	−	cnl
NM-281	−	−	+	−	−	cnl
NM-295	−	+	+	+	+	B
NM-402	−	−	+	+	+	Y
NM-409	−	−	+	−	−	cnl

The prevalence of *N. meningitidis* carriage detected by direct swab PCR was 3.74% (n = 15; 95% CI 2.28–6.08), while the prevalence detected by culture was also 3.74% (n = 15; 95% CI 2.28–6.08). The overall carriage prevalence based on both or either swab PCR or culture was 4.99% (n = 20; 95% CI 3.25–7.58).

### Molecular characteristics of meningococcal carriage isolates

All isolates obtained from the samples that were positive for *porA* but negative for *ctrA* by direct swab PCR were capsule null (*cnl*) and had the *cnl* gene (Table 2). Isolates from two swabs (NM-115 and NM-168) were identified as nongroupable (NG) because they were negative for all genogroup-specific assays, but had the *ctrA* gene and were *cnl* negative (Table 2).

Out of the 15 carriage isolates, 46.7% (n = 7) were *cnl*, followed by genogroups B (26.7%, n = 4), Y (13.3%, n = 2), and NG (13.3%, n = 2). All isolates were grouped into 14 STs, of which 4 were new STs. The isolates were assigned to nine CCs (Table 3). Two genogroup Y isolates were assigned to ST-865 (CC865) and ST-1664, which was not associated with any CCs (UA). *Cnl* was associated with CC53 (n = 1), CC198 (n = 1), and CC1136 (n = 2). Serogroup B isolates were CC269 (n = 2), CC213 (n = 1), and CC4821 (n = 1). The carried serogroup B strains assigned to CC269 were similar to invasive CC269 strains associated with IMD in many countries [24].

**Table 3:** Molecular characteristics of *N. meningiditis* carriage isolates recovered in Lithuania (December 2021–February 2023), and estimation of 4CMenB and MenB-Fhbp vaccine coverage

No of isolates	Genogroup	ST	CC	FetA-VR	PorA		Peptide variant		Vaccine coverage	
					VR1	VR2	fHbp	NHBA	4CMenB	MenB-Fhbp
1	B	213	213	F5-5	22	36	45	18	not covered by fHbp + NHBA	covered
2	B	10864	269	F1-5	19	15−1	13	10	insufficient data	cross-reactive
1	B	17028	4821	F3-36	12−6	13–22	16	669	insufficient data	cross-reactive
1	cnl	53	53	F1-2	7	30	102	58	insufficient data	insufficient data
1	cnl	198	198	F5-5	18	−	−	10	NHBA cross-reactive	not covered
1	cnl	823	198	−	18	25−14	4	10	fHbp + NHBA cross-reactive	cross-reactive
1	cnl	1136	1136	F5-8	18−4	25	−	145	insufficient data	not covered
1	cnl	6096	1136	F5-8	18−4	25	−	145	insufficient data	not covered
1	cnl	11563	11563	F3-8	21−2	23	18	1524	insufficient data	insufficient data
1	cnl	17519	UA	F3-7	17−1	23−7	21	NA	not covered	cross-reactive
1	NG	16804	1157	F5-36	21−7	16	13	114	insufficient data	cross-reactive
1	NG	5953	UA	F5-7	18−1	−	22	601	insufficient data	insufficient data
1	Y	865	865	F1-6	7−1	1	19	19	insufficient data	insufficient data
1	Y	16644	UA	F1-66	7−1	1	321	9	insufficient data	insufficient data

One isolate was negative for the *fetA* gene by PCR, while the PorA VR2 variant was not detected in the two isolates. Vaccine antigens were analyzed in both MenB and non-MenB isolates (Table 3). The 4CMenB vaccine VR2:4 variant was not found in any of the carriage isolates. Three *cnl* isolates were negative for the *fHbp* gene. A total of 10 different fHbp variants were detected, with variant 13 (associated with B and NG genogroups) being the most common (n = 3). Ten variants of the NHBA peptide were found in 14 carriage isolates, with only a partial *nhba* gene sequence available for one *cnl* isolate. The most common NHBA peptides were variant 10 (n = 4) and variant 145 (n = 2). Thirteen isolates lacked the *nadA* gene, while two isolates contained the gene with the encoded NadA-4/5 variant having a frameshift mutation resulting in a premature stop codon in the gene (Additional file 1).

The predicted coverage for all carriage isolates by both MenB vaccines was estimated using the MenDeVAR Index method [30]. The fHbp peptide 45 included in the MenB-Fhbp vaccine had one MenB carriage isolate (Table 3). The peptides 4, 10, 13, and 21 detected in MenB, *cnl*, and NG isolates were considered cross-reactive (‘amber’ category of the MenDeVAR Index). Three MenB and eight non-MenB isolates had insufficient data for the assessment of coverage by the 4CMenB vaccine (‘grey’ category). In total, two isolates were potentially covered by the 4CMenB vaccine and seven by the MenB-Fhbp vaccine (Table 3, Additional file 1).

### Antimicrobial susceptibility of meningococcal carriage isolates

All carriage isolates (n = 15) were susceptible to penicillin (MIC range of < 0.032–0.125 mg/L), ceftriaxone (MIC < 0,016 mg/L), and ciprofloxacin (MIC range < 0.002–0.008 mg/L). One *cnl* isolate was resistant to ciprofloxacin with a MIC of 0.125 mg/L.

### Risk factors associated with meningococcal carriage

A self-reported questionnaire was used to assess the following risk factors: gender, type of residence (apartment/house vs. dormitory), tobacco or electronic cigarette use (none/yes), previous COVID-19 (absent/present), and the frequency of attending parties, bars or clubs in the past two weeks (not attending, 0 times; attending infrequently, 1–3 times; attending frequently, 4 or more times).

The total number of subjects assessed for risk factors was 401. Out of these, 381 (95.0% CI 92.4–96.7%) were non-carriers and 20 (5.0% CI 3.3–7.6%) were carriers of meningococci. The distribution of categories for all carriers and non-carriers is presented in Additional file 2. A few participants did not provide responses to at least one question: one case (0.2%) regarding the place of residence, two cases (0.5%) regarding smoking status, and 10 cases (2.5%) regarding party, bar, and nightclub attendance were missing. The sample size in the univariate regression varies as shown in Additional file 2. In total, there were 388 cases with complete information (96.8%) including 369 non-carriers and 19 carriers, which were included in the multivariate logistic regression.

The findings of the univariate logistic regression analysis suggest that smoking is associated with a higher risk of meningococcal carriage (unadjusted OR = 2.63, CI 1.03–6.55, *p* = 0.038). However, after adjusting for other factors in the multivariate analysis, no significant association was found (*p* = 0.214). Both unadjusted and adjusted logistic regression analyses revealed a significant association between frequent attendance of parties, bars, or clubs (compared to nonvisitors) and *N. meningitidis* carriage status. The unadjusted odds ratio (OR) was 9.50 (95% confidence interval [CI] 2.09–43.4, *p* = 0.003), and the adjusted OR was 8.71 (95% CI 1.82–41.8, *p* = 0.005). However, no significant association was observed between carriage and gender, type of residence, or previous COVID-19. (Additional file 2).

## Discussion

Our study on *N. meningitis* carriage was conducted after the lifting of restrictions due to the COVID-19 pandemic. Return of communities to their prepandemic social behavior has been linked to an increase in IMD cases in other regions [39]. However, this trend has not been observed in Lithuania, and the incidence of IMD remained nearly the same as in 2020–2021.

The present study revealed that the overall prevalence of *N. meningitidis* carriage among university students aged 18–25 (the majority being 20–24 year-olds) was 5%. This prevalence rate is considered low and may correspond to a period of low incidence of IMD in Lithuania. Previous studies, in other countries, have shown varying rates of meningococcal carriage, most commonly ranging from 5 to 10%. A study conducted in Italy during a non-endemic period in 2012–2013 showed a carriage prevalence of 7% among 14–19 year-olds [40]. In the UK, the prevalence of meningococcal carriage in adolescents (aged 15–19 years) during a low IMD incidence period (2014-15) was less than half of that in a comparable population during a high incidence period (1999–2001), accounting for 7.23% and 16.6–18.7%, respectively [41]. In Sweden, the rate of *N. meningitidis* carriage among high school students was 9% [7]. In Canada, a higher carriage prevalence of 29% was detected among university students (18–25 year-olds) in 2010–2013 [5]. Similarly, in the Netherlands, the prevalence was higher among 13–23 year-olds in 2013–2014, with a carriage rate of 16% [4]. In Norway, the carriage rate among 18-year-olds in 2018–2019 was 16.4% [11].

The age group of university students included in our study generally corresponds to the group with the highest carriage prevalence in industrialized countries [3]. However, our study has a limitation in that only a small proportion of 18-year-olds (0.75%) and 19-year-olds (5.2%) were included, which could cause a potentially underestimated prevalence rate of carriage in the overall study population. It is worth noting that enrolling upper secondary school students in any clinical trial in Lithuania is a challenging task, as individuals under the age of 18 require the consent of both parents/guardians [42].

Among the risk factors for meningococcal carriage, frequent attendance at parties, bars, or clubs was found to be associated with the carriage. This finding is consistent with previous research [6, 7]. However, smoking, living in dormitories, and previous COVID-19 were not found to be associated with *N. meningitidis* carriage in our study.

Most isolates of the meningococcal carriage were not encapsulated (*cnl*). *Cnl* strains associated with CC198 and CC1136 in this study were also prevalent in other countries [11, 40]. The limitation of our study was that phenotypic serogrouping of *N. meningitidis* carriage isolates was not performed. MenB was responsible for most IMD cases from 2009 to 2022 in Lithuania, while it accounted for 26.7% of the carriage isolates. Among IMD isolates from 2009 to 2019, MenC was the second most dominant serogroup (7.5%) [18]. However, this serogroup was not found among either invasive or carriage isolates during the present study. Serogroup Y was detected in 2017 and 2019 and constituted approximately 1% (n = 3) of invasive isolates recovered from 2009 to 2019 [18]. The relatively low total number of carriage isolates presents a challenge in determining whether carried MenY strains could be indicative of a potential future increase in IMD cases caused by this particular serogroup in Lithuania.

The encapsulated carriage isolates belonging to CC213 and CC269 were mainly associated with invasive *N. meningitidis* stains reported in Lithuania or other countries [24]. Although MenB CC32 was prevalent among recovered invasive isolates of *N. meningitidis* which accounted for 74% of all invasive meningococcal isolates recovered from 2009 to 2019 [18] and 64.7% from 2020 to 2022, it was not detected among carriage isolates. This result is in accordance with data collected in Norway over a 12-year period, where the detected carriage rate of CC32 was low (about 5%) for the whole time, although this CC was responsible for 70% of IMD cases in 1991 and 15% in 2003 [1]. Our data confirm that the carriage rate of hyperinvasive strains is not directly related to the incidence of IMD [1]. We may speculate that the absence of CC32 among the carriage strains is a consequence of vaccination against MenB. However, there is no evidence for a reduction in serogroup B and other serogroups carriage density induced by the MenB vaccine [16] in contrast to the effect of vaccine against serogroup C meningococci [15]. The mechanism underlying the lack of impact of the MenB vaccine on the carriage is not yet fully understood [43].

We analyzed serogroup B vaccine antigens in MenB and non-MenB carried meningococci to better understand the carrier state. The vaccine antigens were present in non-MenB meningococci and may protect against non-MenB-caused IMD. Only two *cnl* isolates, retained vaccine antigens that were considered cross-reactive and predicted to be covered by the 4CMenB vaccine. Nearly one-half of the isolates were potentially covered by the Men-Fhbp vaccine.

## Conclusions

This study represents the first investigation of *N. meningitidis* carriage in Lithuania. The prevalence of carriage, which was 5% among university students aged 18–25, is generally consistent with rates reported in other European countries. The carriage isolates showed genetic diversity, although a large proportion was identified as capsule null locus. Studies on *N. meningitidis* carriage revealed that monitoring the carriage prevalence including the molecular characteristics of strains would be advantageous for understanding the epidemiology of IMD and for evaluating the impact of vaccination programmes which are important for controlling the disease in the country.

### Electronic supplementary material

Below is the link to the electronic supplementary material.

Supplementary Material 1

Supplementary Material 2

## Data Availability

The datasets analyzed during the current study are available from the corresponding author upon request.
