# Supplementary material for: Molecular characteristics of Neisseria meningitidis carriage strains in university students in Lithuania
Source: BMC Microbiol. 2023 Nov 17;23:352. doi: 10.1186/s12866-023-03111-5 (PMC10655475; doi:10.1186/s12866-023-03111-5)
Supplement: Supplementary file 2 — Supplementary Material 2 [file 12866_2023_3111_MOESM2_ESM.pdf]

Univariate and multivariate logistic regression analysis of risk factors for *N. meningitidis* carriage

		Unadjusted			Distribution of cases (used in univariate analyses)			Adjusted			Distribution of cases (used in multivariate / adjusted analysis)	
**Characteristic**	**N**[1]	**OR**	**95% CI**	***p***[2]	**Negative** N = 381	**Positive** N = 20	**N**	**OR**	**95% CI**	***p***	**Negative** N = 36[3]	**Positive** N = 19
**Gender**	401						388					
Female	*(ref)*[3]	—	—		297 (78%)	13 (65%)	*(ref)*	—	—		286 (78%)	13 (68%)
Male		1.90	0.70, 4.80	0.184	84 (22%)	7 (35%)		1.52	0.50, 4.13	0.431	83 (22%)	6 (32%)
**Type of residence**	400						388					
Apartment/house	*(ref)*	—	—		298 (78%)	13 (68%)	*(ref)*	—	—		288 (78%)	13 (68%)
Dormitory		1.66	0.57, 4.33	0.321	83 (22%)	6 (32%)		1.78	0.59, 4.83	0.272	81 (22%)	6 (32%)
Unknown[4]					0	1						
**Smoking**	399						388					
No	*(ref)*	—	—		289 (76%)	11 (55%)	*(ref)*	—	—		282 (76%)	11 (58%)
Yes		2.63	1.03, 6.55	**0.038**	90 (24%)	9 (45%)		1.87	0.67, 4.95	0.214	87 (24%)	8 (42%)
Unknown					2	0						
**Previous COVID-19**	401						388					
No	*(ref)*	—	—		188 (49%)	10 (50%)	*(ref)*	—	—		180 (49%)	10 (53%)
Yes		0.97	0.39, 2.43	0.954	193 (51%)	10 (50%)		0.80	0.30, 2.11	0.650	189 (51%)	9 (47%)
**No. of party/bar/ night club visits**	391						388					
0	*(ref)*	—	—		171 (46%)	4 (20%)	*(ref)*	—	—		169 (46%)	4 (21%)
1-3		2.82	0.96, 10.2	0.078	182 (49%)	12 (60%)		2.31	0.75, 8.59	0.167	182 (49%)	11 (58%)
4 or more		9.50	2.09, 43.4	**0.003**	18 (4.9%)	4 (20%)		8.71	1.82, 41.8	**0.005**	18 (4.9%)	4 (21%)
Unknown					10	0						

[1] N, number of non-missing values
[2] Statistically significant *p*-values (p<0.05) are in red.
[3] Reference group
[4] Unknown values were not included in the logistic regression
